# Efficacy of Intraoperative Mitomycin-C in Vasovasostomy Procedure: A Randomized Clinical Trial 

**DOI:** 10.22074/ijfs.2019.5664

**Published:** 2019-07-14

**Authors:** Farzad Allameh, Jalil Hosseini, Hamidreza Qashqai, Hamzeh Mazaherylaghab

**Affiliations:** 1 Urology and Nephrology Research Center, Shahid Beheshti University of Medical Sciences, Tehran, Iran; 2 Men's Health and Reproductive Health Research Center (MHRHRC), Reconstructive Urology Department, Shohada-e-Tajrish Hospital, Shahid Beheshti University of Medical Sciences, Tehran, Iran; 3 Urology Department, Imam Sajjad Hospital, Iran University of Medical Sciences, Shahriar, Iran; 4 Faculty of Medicine, Hamedan University of Medical Sciences, Hamedan, Iran

**Keywords:** Clinical Trial, Mitomycin C, Sperm Count, Vasectomy Reversal, Vasovasostomy

## Abstract

**Background:**

Two-six percentage of vasectomized men will ultimately seek vasectomy reversal, which late stricture and obstruction after operation are relatively common. To find a method for improving vasovasostomy outcomes, we used intra-operative local mitomycin-C (MMC) preventing possible fibrosis and stricture.

**Materials and Methods:**

In this randomized clinical trial, 44 patients were assigned to two groups randomly during a one-year study and the data of 40 patients were analyzed. The patients were followed up for 6 months after surgery. The case group (n=19) was treated by vasovasostomy with intra-operative local MMC. The control group (n=21) underwent standard vasovasostomy.

**Results:**

Mean sperm count in MMC group was significantly higher than the controls. The sperm count of more than 20 million/ml was respectively 53% and 14% in MMC and control groups. In a subgroup where the interval between vasectomy and reversal was 5-10 years, post-reversal azoospermia was absent in MMC group, but 50% of the controls were still azoospermic. In addition, 80% of MMC group had more than 20 million/ml sperms, but all of the controls had less than 20 million/ml sperms. No significant complication was seen.

**Conclusion:**

Intra-operative local MMC in vasovasostomy can be regarded as a safe and efficient technique which has several advantages including lower cost. Increase of sperm count is the main effect of local MMC applica- tion that is more prominent when the interval between vasectomy and reversal is 5-10 years (Registration number: IRCT2015092324166N1).

## Introduction

Approximately 6-8% of married couples (Fig about 42-60 million men), experience vasectomy as contraception (1). Surveys suggest that 2-6% of vasectomized men will ultimately seek for vasectomy reversal (2). The most common indications for vasectomy reversal are divorce, death of spouse or child and relief from post-vasectomy pain syndrome (3).

A meta-analysis on 32 studies about vasovasostomy with 6633 patients revealed that mean post-procedure patency and pregnancy rates were 89.4 and 73.0%, respectively, with the mean obstruction interval of 7.2 years. No statistically significant difference in vasovasostomy outcomes was seen in the comparison of single versus multilayer anastomosis. Obstructive interval less than 10 years was a predictor of higher patency and pregnancy rates (4). Other analyses and studies had less patency or pregnancy rates, 60-86% and 25- 53%, respectively (5-7). The main predictors for success of the reversal procedure were the time between vasectomy and reversal, as well as female partner age (6, 8). History of conception with the current partner versus remarriage (7), average testicular volume (9), presence of a sperm granuloma, use of surgical clips instead of suture at vasectomy, presence and quality of vasal fluid and sperm in vasal fluid during surgical exploration, in addition to increased α-glucosidase in the postoperative semen also had a favorable impact on patency (5, 10). Some studies reported that smoking of the male or female partner and obstructive interval did not correlate with postoperative success (7, 11).

The most common early complication of vasovasostomy is hematoma. The hematomas are perivasal and very small, thus they usually require no surgical drainage. Wound infection is another possible early complication. Late complications include sperm granuloma at the anastomotic site (5%). Late stricture and obstruction are relatively common (12- 18% in 12 months). With microsurgical techniques, patency can reach to 70-90% (12). Some newer techniques are introduced to obtain better results including laser tissue soldering (13), angled cutting for increasing vasal surface area, increasing neovascularity and decreasing fibrosis (14), using a double-ringed instrument designed to facilitate handling and dissecting vas away from perivasal tissue in an atraumatic fashion (15) and application of the fibrin glue (16).

Several surgeons have used mitomycin-C (MMC) as an antifibrotic adjunct to ab-externo trabeculectomy and Dacryocystorhinostomy (DCR). It seems that intra-operative local MMC with a controlled concentration is a safe agent for reducing fibrosis (17, 18). MMC is an antimitotic and cytotoxic agent that crosslinks DNA. This agent inhibits DNA synthesis, cellular RNA synthesis and nuclear division. MMC also induces apoptosis and inhibits protein synthesis by hampering synthesis of the collagen using fibroblasts (19-22). In animal models, studies on grafted tissue in mice have revealed that the differentiation of grafts was significantly inhibited by MMC (23). In human studies, fibroblasts showed a dramatic structural response to MMC, including intracellular edema, pleomorphic and vesicular mitochondria changes, dilated smooth and rough endoplasmic reticulum, as well as chromatin condensation (24).

Evidence for MMC-induced carcinogenicity is considered sufficient for animals, but inadequate for humans. As such, MMC is classified by International Agency for Research on Cancer (IARC) as possibly carcinogenic agent to humans (group 2B). A meta-analysis studied the effect of varying concentrations of MMC and treatment durations on cellular proliferation and viability of the fibroblasts. They found MMC at 0.4 mg/ml beyond the 5 minutes, and 0.5 mg/ml concentration at all time-points were lethal and caused extensive cell deaths, compared to controls. The minimum effective concentration appeared to be 0.2 mg/ml for 3 minutes (25). In a systematic review, it was found that intra-operative MMC adjunct in trabeculectomy appears to reduce the relative risk of failure, and no significant increase in permanent sight-threatening complications was detected. They reported that MMC was administered intra-operatively in concentrations of 0.1-0.5 mg/ml concentrations of saline for durations varying from 1-5 minutes (26). Local injection of MMC in the site of Internal Ureterotomy (IU) was also studied by several groups, reported that submucosal MMC injection reduced the stricture rate from 50% to 10%, after IU (27). 

The important point is that all of the previous studies have examined MMC as an anti-fibrotic agent for ophthalmologic surgeries and internal urethrotomies. But intra-operative local MMC has not been studied in vasovasostomy yet. Therefore, our study is performed to determine the overall safety and efficacy of intra-operative local MMC as the anti-fibrotic agent in vasovasostomy.

## Materials and Methods

In this randomized clinical trial, 58 patients, visited for vasectomy reversal in Shohada-e-Tajrish Hospital (Tehran, Iran) between January and October 2016, were enrolled. 

### Patient and public involvement statement

The main priority of these patients was to have the opportunity of becoming a father. It was indicated to the patients that this method may not improve the outcome of vasovasostomy procedure and they preferred to participate in this trial. All patients were fully informed about the method of trial and subsequently they were blindly sub-grouped. All recruited and conducted participants were informed about the trial results by email after data analysis.

In this randomized controlled trial (RCT) the burden of the intervention such as pain and surgical site infection, or hematoma were assessed by patients and also residents of urology in the outpatient clinic and they were then recorded in our database.

Inclusion criterion was ‘males who underwent vasectomy and wanted reversal of vasectomy. Exclusion criteria were testicular atrophy, history of urethral or bladder neck surgery, history of previous vasovasostomy, history of scrotal region radiotherapy, history of chemotherapy, age of partner out of fertility range and any situation suggesting the need for vasoepididymostomy.

Six patients had testicular atrophy, history of previous vasovasostomy and age of their partners was out of fertility range. Eight patients were candidates for vasoepididymostomy, because of previous scrotal surgery or manipulation like percutaneous sperm aspiration (PESA). Hence, all of them were excluded from the allocation.

Finally, 44 consecutive patients were allocated randomly into two groups: the case group (n=22) was candidate for vasovasostomy in addition to intra-operative local MMC. The control group (n=22) was allocated for standard vasovasostomy. Randomization was performed by a random number table and opaque envelopes were used for allocation.

The primary endpoints included presence of sperm in semen, sperm count more than 20 million/ml, sperm motility rate and normal morphology rate in sperms. The secondary endpoints include hematoma, inflammatory reaction, tissue necrosis and any sign of surgical site infection. As mentioned before, all patients were informed about the disease, method of study and treatment possibilities. They had been informed about the possible complications and other applicable managements. Then, an informed consent was taken from each patient.

The proposal of this study was approved by Shahid Beheshti Medical University (SBMU) Ethical Committee (IR.SBMU.MSP.REC.1395.100) and research board of Infertility and Reproductive Health Research Center (IRHRC). Ethical issues were respected based on Declaration of Helsinki. The RCT was approved and documented by IRCT (IRCT2015092324166N1).

Initial pre-operative evaluations included detailed medical history, complete physical examination and sperm analysis. In MMC group, pre-operation evaluation included laboratory tests and cardiovascular consultation. In the operating room, under spinal anesthesia, the procedure was carried out using bilateral high vertical incision of scrotum. After finding each vas deferens and preparing the site of anastomosis, two ends of vas deferens were floated in 0.2 mg/ml MMC solution for 5 minutes, and they were then washed by normal saline. Finally, anastomosis was performed microscopically (CARL ZEISS F170 T surgical microscope binoculars 10×/22B; Zeiss, Germany) using modified two-layered vasovasostomy. Two 5-0 poly-propylene sutures were placed at 5 and 7 o’clock positions in the sero-muscular layer to approximate two ends of the vas. Next, four 8-0 poly-propylene sutures were sequentially placed inside out in the mucosa of the vasal ends, at 3, 6, 9, and 12 o’clock positions and tied up. Two additional sero- muscular sutures were placed at 1 and 11 o’clock positions to complete the anastomosis. In the control group, vasovasostomy procedure was carried out as the MMC group, except for floatation in MMC solution. All surgeries were performed by the same surgical team.

Upon finishing the procedure, patients in both groups were in complete bed rest the day after operation. The second day after surgery, they were discharged providing the tests and general condition were normal. Patients were advised to have relative rest at home for two weeks, avoiding intercourse for one month and to have scrotal support for at least one week. The patients were informed about possible early and late complications, in addition to the time of next necessary following up visits. The patients were followed up at 1, 3, and 6 months after surgery by a complete history and a physical examination to monitor the complications (hematoma, inflammatory reaction, tissue necrosis and any sign of operation failure). Sperm analysis was also performed 1 and 6 months after surgery for measuring patency (presence of sperm in semen), sperm count, sperm morphology and motility.

**Fig 1:**
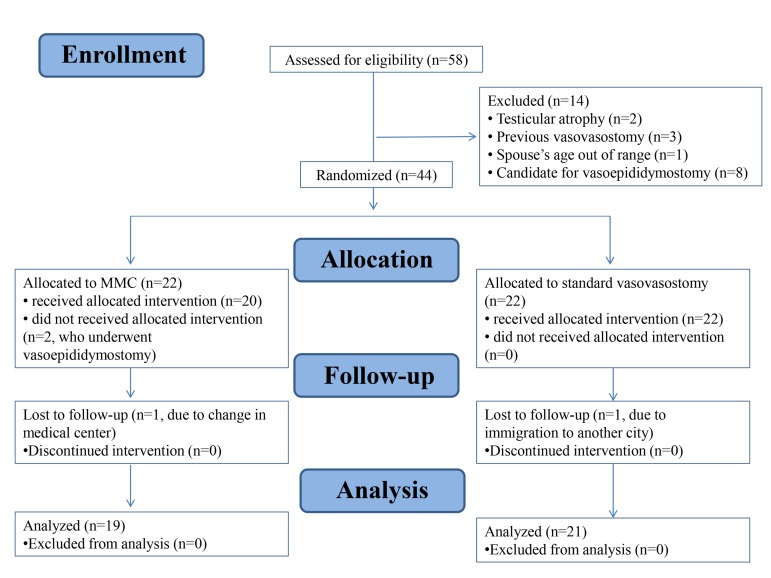
CONSORT 2010 flow-diagram.

These data were gathered and documented via checklists consisting demographical data which include the interval between vasectomy and vasovasostomy, intra- operative local MMC application, sperm analysis results and any complication related to the procedure. In MMC group, during the procedure, two patients were not compatible with the inclusion criteria, since they were candidate for vasoepididymostomy. So, they were omitted from the study and 20 patients received allocated intervention. In this group one patient lost the follow up. Finally, the data of 19 patients were analyzed. In the control group, all of the 22 patients received allocated intervention. During follow up, one patient immigrated to another city and he was out of reach. Therefore, the data of 21 patients were analyzed. Figure 1 shows the CONSORT flow-diagram of the data in this study. The data analysis method was per- protocol and performed by SPSS (version 23.0) software (SPSS, Chicago, USA). Fisher exact test, Independent t test, chi-square test and likelihood ratio chi square test were used to compare and analyze the data. P value significance level was defined as 0.05.

**Table 1:** Primary data analysis


Group	Mean age (Y)	Normalmorphology (%)	Motile sperms (%)	Sperm count		Mean sperm count (m/ml)	Patency	
				<20 M/ml	>20 M/ml		Azoospermia	Sperm present

MMC	39.95 ± 5.553	20.05 ± 14.69	27.05 ± 16.98	9 (47)	10 (53)	(23.6 ± 2.3)×10^6^	4 (21)	15 (79)
Control	40.95 ± 6.659	17.05 ± 17	18.71 ± 15.96	18 (86)	3 (14)	(9.4 ± 1.4)×10^6^	9 (43)	12 (57)
P value	0.609	0.559	0.118	0.017		0.023	0.186	


Data are presented as mean ± SD or n (%). MMC; Mitomycin-C.

**Table 2:** Data analysis based on post-vasectomy interval


Group	Patency		Sperm count	
	Sperm present	Azoospermia	>20 M/ml^*^	<20 M/ml

Interval<5 Y (n=7)				
MMC	2 (50)	2 (50)	0	4 (100)
Control	3 (100)	0	1 (33)	2 (67)
P value	0.092		0.166	
5 Y<interval<10 Y (n=18)				
MMC	10 (100)	0	8 (80)	2 (20)
Control	4 (50)	4 (50)	0	8 (100)
P value	0.005		0.0001	
Interval>10 Y (n=15)				
MMC	3 (60)	2 (40)	2 (40)	3 (60)
Control	5 (50)	5 (50)	2 (20)	8 (80)
P value	0.714		0.417	


Data are presented as n (%). *; Likelihood ratio chi square test, MMC; Mitomycin C, and Y; Year.

## Results

Mean age in MMC group and control group was 39.95 (± 5.55) and 40.95 (± 6.65) years, respectively (P=0.609, Table 1). There was no early or late surgical complication in our allocated patients. Six months after surgery, mean sperm motility in MMC and the control group was identical (27.05 and 18.71% respectively, P=0.118). Normal morphology rate was also the same (20.05 and 17.05% respectively, P=0.559) (Table 1). Mean sperm count in MMC group was higher than the controls (23.5 and 9.4 million/ml) (P=0.023), and sperm count more than 20 million/ml in MMC and the control group was 53 and 14%, respectively (P=0.017). These differences were significant, but post reversal azoospermia in the two groups was not different (21% in MMC group and 43% in controls, P=0.186) (Table 1).

Then, we analyzed data in three subgroups based on the interval between vasectomy and reversal (less than 5, 5-10 and more than 10 years). In the first subgroup (less than 5 years interval), post reversal azoospermia (P=0.429) and sperm count more than 20 million/ml (P=0.429) in MMC and control groups were not statistically different. In the second subgroup (5-10 years interval), post reversal azoospermia was absent in MMC group, but 50% of the controls were still azoospermic (P=0.023). In addition, 80% of MMC group had more than 20 million/ml sperms, but all of the controls had less than 20 million/ml sperms (P=0.001). In the third subgroup (more than 10 years of interval), there was no statistical difference in post reversal azoospermia (P=1.000), and sperm count more than 20 million/ml (P=0.560) in the two groups (Table 2).

## Discussion

Intra-operative MMC application is described for DCR, trabeculectomy, and some urological surgeries. All of these reports emphasized that MMC, as a local antifibrotic agent, is effective and safe. This trial, for the first time, demonstrates the effects of local intra-operative MMC in vasovasostomy. We cannot use previous trial estimate the best sample size. So we conducted a pilot study to find if any benefit exist using intra-operative MMC in vasectomy reversal. It seems that the increase of sperm count is the main effect of local intra-operative MMC in vasovasostomy, but it has no effect on sperm motility and morphology. This effect is more prominent in both patency and sperm count more than 20 million/ ml; especially, in a subgroup with 5-10 years of interval between vasectomy and reversal. If the interval is less than 5 years or more than 10 years, MMC application has no benefit in the reversal outcomes. It is important that MMC application has lower cost in comparison with intracytoplasmic sperm injection (ICSI) or other new techniques described for vasovasostomy, and it has no side effects if the concentration is controlled. It needs no special training and the time of surgery is relatively the same as standard vasovasostomy.

The main limitations of our study are small sample size, the use of very low concentration of MMC, relatively short follow up term and not enough follow up to study the pregnancy rate.

## Conclusion

Intra-operative local MMC in vasovasostomy can be regarded as a safe and efficient technique which has several advantages including lower cost. Increase of sperm count is the main effect of local MMC application that is more prominent when the interval between vasectomy and reversal is 5-10 years. However, further studies should be conducted with larger sample sizes and different MMC dosage, longer durations, and multi-center sampling to attain more definite results.
